# Augmented reality in interventional radiology education: a systematic review of randomized controlled trials

**DOI:** 10.1590/1516-3180.2021.0606.R2.27122021

**Published:** 2022-08-08

**Authors:** And Yara Particelli Gelmini, Márcio Luís Duarte, Mayara Oliveira da Silva, Josias Bueno Guimarães, Lucas Ribeiro dos Santos

**Affiliations:** I MD. Radiologist, Department of Radiology, WEBIMAGEM Telerradiologia, São Paulo (SP), Brazil.; II MSc. Musculoskeletal Radiologist, Centro Radiológico e Especialidades Médicas São Gabriel, Praia Grande (SP), Brazil; and Doctoral Student in Evidence-based Health Program, Universidade Federal de São Paulo (UNIFESP), São Paulo (SP), Brazil.; Universidade Federal de São Paulo, Evidence-based Health Program, São Paulo, SP, Brazil; III BSc. Biomedic, Biomedical imaging, Clínica Mega Imagem, Santos (SP), Brazil.; IV B.Eng. Engineer, Department of Engineering, MIP Engenharia, Belo Horizonte (MG), Brazil.; V MSc. Endocrinologist, Department of Physiology and Medical Clinic, and Professor of Physiology and Medical Clinic, Centro Universitário Lusíada (UNILUS), Santos (SP), Brazil; and Doctoral Student in Evidence-based Health Program, Universidade Federal de São Paulo (UNIFESP), São Paulo (SP), Brazil.; Universidade Federal de São Paulo, Evidence-based Health Program, São Paulo, SP, Brazil

**Keywords:** Radiology, interventional, Augmented reality, Education, medical, Simulation training, Video game, Perk Tutor, Google Glass, Wearable technology

## Abstract

**BACKGROUND:**

Augmented reality (AR) involves digitally overlapping virtual objects onto physical objects in real space so that individuals can interact with both at the same time. AR in medical education seeks to reduce surgical complications through high-quality education. There is uncertainty in the use of AR as a learning tool for interventional radiology procedures.

**OBJECTIVE:**

To compare AR with other learning methods in interventional radiology.

**DESIGN AND SETTING:**

Systematic review of comparative studies on teaching techniques.

**METHODS:**

We searched the Cochrane Library, MEDLINE, Embase, Tripdatabase, ERIC, CINAHL, SciELO and LILACS electronic databases for studies comparing AR simulation with other teaching methods in interventional radiology. This systematic review was performed in accordance with PRISMA and the BEME Collaboration. Eligible studies were evaluated using the quality indicators provided in the BEME Collaboration Guide no. 11, and the Kirkpatrick model.

**RESULTS:**

Four randomized clinical trials were included in this review. The level of educational evidence found among all the papers was 2B, according to the Kirkpatrick model. The Cochrane Collaboration tool was applied to assess the risk of bias for individual studies and across studies. Three studies showed an improvement in teaching of the proposed procedure through AR; one study showed that the participants took longer to perform the procedure through AR.

**CONCLUSION:**

AR, as a complementary teaching tool, can provide learners with additional skills, but there is still a lack of studies with a higher evidence level according to the Kirkpatrick model.

**SYSTEMATIC REVIEW REGISTRATION NUMBER:**

DOI 10.17605/OSF.IO/ACZBM in the Open Science Framework database.

## INTRODUCTION

Learning is the process of acquiring new knowledge and skills, and this process has its difficulties and pitfalls.^1,2^ In medicine, acquiring new abilities can lead to improvement in outcomes, as in the field of surgery, in which open surgical procedures have been replaced by minimally invasive procedures, and fresh devices are created to refine surgical abilities, and teaching processes as well.^3,4^

The “learning before doing” concept is rapidly replacing the conventional “see one, do one, teach one” technique, in order to avoid potential mistakes.^5,6^ According to British National Health Service data, preventable injuries and deficient medical training are responsible for 10% of hospitalizations.^7^ In consonance, “warm-up” can be applied to students and experienced professionals, thus boosting performance and self-confidence.^8^ This could form another application for augmented reality (AR).

AR involves digitally overlapping virtual objects onto physical objects in real space so that individuals can interact with both at the same time. Virtual reality produces immersion of the user in a given environment, which may or may not be controlled, by depriving the perception of the local environment through use of a computerized scenario or one previously captured on video, and experiencing an environment as if it existed.^9–15^ With AR, users visualize the real situation in which they are immersed along with a virtual projection of a 3D image. This immersion can be enhanced with sound, touch and smell through integrated external components.^10,11,13,16–18^ Increasingly, use of mobile AR (mAR) makes time and location flexible and expands training time.^10,19,20^

Interventional radiology consists of imaging-guided minimally invasive procedures that enable lower morbidity and shorter hospitalization time.^7^ Spatial and cognitive proprioception are the main difficulties identified during training.^21,22^ Acquisition of skills to use new devices is also a common issue, which can cause tragic outcomes, especially at the start of a career.^21,22^ Therefore, AR may improve medical teaching and enhance skills relating to given procedures.^23,24^ Preliminary studies comparing use of AR with traditional teaching methods have produced promising results.^3,4,25^

There is no systematic review about augmented reality in interventional radiology.

## OBJECTIVE

The aim of this study was to identify, systematically analyze and summarize the best available evidence comparing AR teaching techniques with various other methods in interventional radiology.

## METHODS

The PICO technique (Population, Intervention, Comparison, Outcome) was used to define the study, as follows:

P = Undergraduate healthcare students; postgraduate trainees; continuous professional development training – independent of the specialties.

I = Augmented reality to teach interventional radiology.

C = Traditional methodology versus AR.

O = Improve ultrasound skills to achieve an accurate diagnosis

### Study model

This systematic review was executed in accordance with the Preferred Reporting Items for Systematic Reviews and Meta-Analyses (PRISMA) and Best Evidence Medical Education (https://www.bemecollaboration.org/), and was registered in the Open Science Framework (https://osf.io/wn762). This study was exempted from institutional review as no live subjects were studied.

### Inclusion criteria

We included studies that compared the AR method with several other teaching methods – phantom, cadavers, porcine method and didactic teaching (books, articles, lectures without the use of AR) – in interventional radiology. No restrictions concerning the language, publication status of the study or population were imposed.

### Selection of studies and data extraction

Eligible studies were identified using a two-stage method by two independent reviewers (AYPG, MLD). Disagreements were settled by reaching a consensus. First, after eliminating duplicates, titles and abstracts retrieved through the search strategy were evaluated, thus yielding potentially eligible studies. Second, full-text evaluation of the pre-selected studies was performed to confirm eligibility; this process was carried out through the Rayyan platform (https://rayyan.qcri.org).^26^

### Evaluation of methodological quality

The Cochrane Collaboration tool was applied to assess the risk of bias in individual studies and across studies.^27^ Eligible randomized controlled trials (RCTs) were analyzed using the quality indicators from Best Evidence Medical Education (BEME) Collaboration Guide no. 11^28^ (Annex 1) and the Kirkpatrick model (BEME Guide no. 8) (Table 1).^29,30^ According to BEME Guide no. 11, higher quality studies meet a minimum of seven out of eleven indicators. The tools are well established and cover a wide spectrum of methodological issues.

**Table 1:** Kirkpatrick’s hierarchy^30^

Level	Feature	Evaluation
1	Reaction	Participants’ opinions about the learning experience, its organization, presentation, content, teaching methods and quality of instruction
2A	Learning - Change in attitude	Changes in attitudes or perceptions among participating groups concerning teaching and learning
2B	Learning - Modification of knowledge or skills	For knowledge, this refers to acquisition of concepts, procedures and principles For skills, this refers to acquisition of thinking/problem-solving, psychomotor and social skills
3	Behavior - Behavioral change	Documents transfer learning to the workplace or students’ willingness to apply new knowledge and skills
4A	Results - Change in the organizational system/practice	Refers to broader changes in the organization, attributable to the educational program
4B	Results - Change between participants, students, residents or colleagues	Refers to the improvement in learning/performance of students or residents as a direct result of educational intervention

Articles that did not compare teaching methods, along with those with a population dropout rate ≥ 50% (as prescribed in BEME Collaboration Guide no. 11) and those analyzing factors other than medical teaching, were excluded.

### Search methods for choosing studies

Electronic searches were performed in the PubMed, Cochrane Library, Embase, ERIC, CINAHL, Tripdatabase and SciELO databases, using the following MeSH terms: Interventional Radiology; Virtual Reality; Augmented Reality; Video Games; Computer Simulation; Education, Medical; Teaching; and Simulation Training.

References from the studies included and from the main reviews on the subject were also analyzed. The search strategies were carried out on July 29, 2020, for each database, and are shown in Table 2.

**Table 2:** Search strategy according to the corresponding database

Database	Search strategy
Cochrane Library	#1: MeSH descriptor: [Radiology, Interventional] explode all trees #2: MeSH descriptor: [Virtual Reality] explode all trees #3: MeSH descriptor: [Augmented Reality] explode all #4: MeSH descriptor: [Video Games] explode all trees #5: MeSH descriptor: [Computer Simulation] explode all trees #6: MeSH descriptor: [Education, Medical] explode all trees #7: MeSH descriptor: [Teaching] explode all trees #8: MeSH descriptor: [Simulation Training] explode all trees #9: #1 AND #2 OR #3 OR #4 OR #5 AND #6 OR #7 OR #8
MEDLINE	#1: “Radiology, Interventional”[MeSH] OR (Interventional Radiology) #2: “Virtual Reality” [MeSH] OR (Reality, Virtual) OR (Virtual Reality, Educational) OR (Educational Virtual Realities) OR (Educational Virtual Reality) OR (Reality, Educational Virtual) OR (Virtual Realities, Educational) OR (Virtual Reality, Instructional) OR (Instructional Virtual Realities) OR (Instructional Virtual Reality) OR (Realities, Instructional Virtual) OR (Reality, Instructional Virtual) OR (Virtual Realities, Instructional) OR “Augmented Reality“[MeSH] OR (Augmented Realities) OR (Realities, Augmented) OR (Reality, Augmented) OR (Mixed Reality) OR (Mixed Realities) OR (Realities, Mixed) OR (Reality, Mixed) OR “Video Games”[MeSH] OR (Game, Video) OR (Games, Video) OR (Video Game) OR (Computer Games) OR (Computer Game) OR (Game, Computer) OR (Games, Computer) OR “Computer Simulation”[MeSH] OR (Computer Simulations) OR (Simulation, Computer) OR (Simulations, Computer) OR (Computerized Models) OR (Computerized Model) OR (Model, Computerized) OR (Models, Computerized) OR (Models, Computer) OR (Computer Models) OR (Computer Model) OR (Model, Computer) OR (In Silico) OR (In Silicos) OR (Silico, In) OR (Silicos, In) #3: “Education, Medical”[MeSH] OR (Medical Education) OR “Teaching”[MeSH] OR (Training Techniques) OR (Technique, Training) OR (Techniques, Training) OR (Training Technique) OR (Training Technics) OR (Technic, Training) OR (Technics, Training) OR (Training Technic) OR (Pedagogy) OR (Pedagogies) OR (Teaching Methods) OR (Method, Teaching) OR (Methods, Teaching) OR (Teaching Method) OR (Academic Training) OR (Training, Academic) OR (Training Activities) OR (Activities, Training) OR (Training Activity) OR (Techniques, Educational) OR (Technics, Educational) OR (Educational Technics) OR (Educational Technic) OR (Technic, Educational) OR (Educational Techniques) OR (Educational Technique) OR (Technique, Educational) OR “Simulation Training”[MeSH] OR (Training, Simulation) OR (Interactive Learning) OR (Learning, Interactive) #4: #1 AND #2 AND #3
EMBASE	#1: interventional radiology/exp #2: virtual reality/exp #3: augmented reality/exp #4: video game/exp #5: computer simulation/exp #6: medical education/exp #7: simulation training/exp #8: teaching/exp #9: #1 OR #2 AND #3 OR #4 OR #5 OR #6 AND #7 OR #8
LILACS	#1: mh: “Radiologia Intervencionista” OR (Radiología Intervencional) OR (Radiology, Interventional) OR (H02.403.740.675) #2: mh: “Realidade Virtual” OR (Realidad Virtual) OR (Virtual Reality) OR (Educational Virtual Realities) OR (Educational Virtual Reality) OR (Instructional Virtual Realities) OR (Instructional Virtual Reality) OR (Realities, Instructional Virtual) OR (Reality, Educational Virtual) OR (Reality, Instructional Virtual) OR (Reality, Virtual) OR (Virtual Realities, Educational) OR (Virtual Realities, Instructional) OR (Virtual Reality, Educational) OR (Virtual Reality, Instructional) OR (L01.224.160.875) OR (L01.296.555) OR (SP4.011.127.428.806.030) #3: mh: “Realidade Aumentada” OR (Realidad Aumentada) OR (Augmented Reality) OR (Augmented Reality for Health) OR (Augmented Reality in Clinical Simulations) OR (Augmented Reality in Health Care Education) OR (Augmented Reality in Health) OR (Augmented Reality in Healthcare Education) OR (SP4.011.127.428.806.020) #4: mh: “Jogos de Vídeo” OR (Juegos de Video) OR (Video Games) OR (Computer Game) OR (Computer Games) OR (Game, Computer) OR (Game, Video) OR (Games, Computer) OR (Games, Video) OR (Video Game) OR (I03.450.642.693.930) OR (L01.224.900.930) #5: mh: “Educação de Graduação em Medicina” OR (Educación de Pregrado en Medicina) OR (Education, Medical, Undergraduate) OR (Education, Undergraduate Medical) OR (Medical Education, Undergraduate) OR (Undergraduate Medical Education) OR (I02.358.399.450) #6: mh: “Treinamento por Simulação” OR (Entrenamiento Simulado) OR (Simulation Training) OR (Interactive Learning) OR (Learning, Interactive) OR (Training, Simulation) OR (I02.903.847) #7: #1 AND #2 OR #3 OR #4 AND #5 OR #6
Tripdatabase	(Interventional radiology)(Virtual reality OR Augmented reality OR Video game OR Computer simulation)(Medical education OR Simulation training OR Teaching)
ERIC	#1: Interventional radiology #2: Virtual reality #3: Augmented reality #4: Video game #5: Computer simulation #6: Medical education #7: Simulation training #8: Teaching #9: #1 OR #2 AND #3 OR #4 OR #5 OR #6 AND #7 OR #8
CINAHL	#1: Interventional radiology #2: Virtual reality OR vr OR augmented reality OR video games OR computer simulation #3: Medical education OR simulation training or simulation education or simulation learning OR teaching #4: #1 AND #@ AND #3
SciELO	#1: Interventional radiology #2: Virtual reality #3: Augmented reality #4: Video game #5: Computer simulation #6: Medical education #7: Simulation training #8: Teaching #9: #1 OR #2 AND #3 OR #4 OR #5 OR #6 AND #7 OR #8

## RESULTS

**Figure 1:**
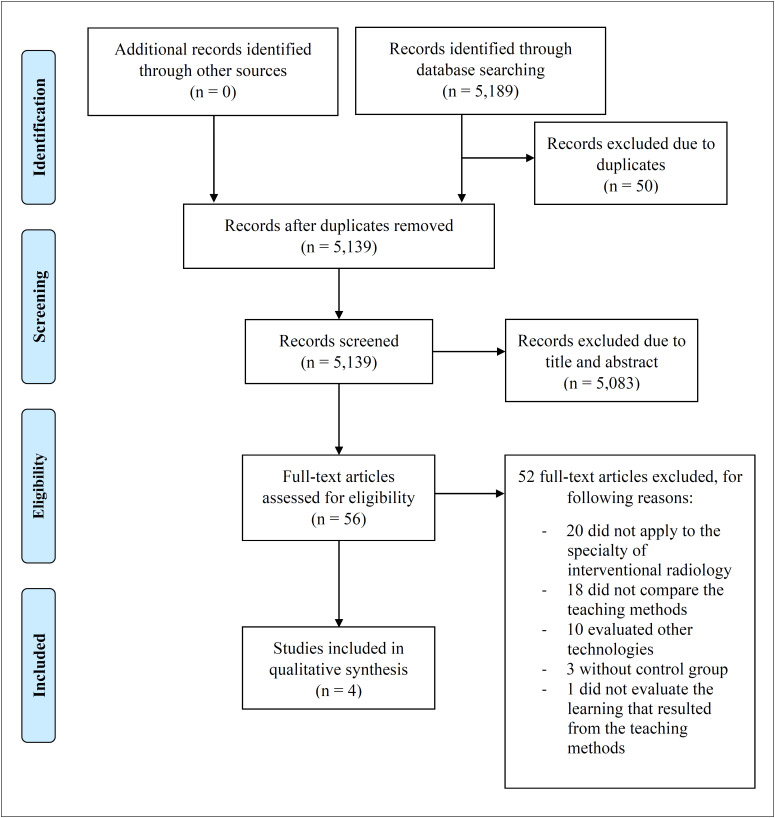
PRISMA flow diagram of study selection.

The search yielded 5189 articles; 50 of these were duplicates and were excluded. Through analysis on the titles and abstracts, 56 articles were selected for full-text evaluation, out of which four were included (Figure 1). Among these 56 articles, Grasso et al.^31^ did not evaluate the learning that resulted from the teaching methods and was excluded from the analysis.

Two of the four studies were from Canada^15,17^ and used a pre-experience questionnaire; the other two were from the United States^14,18^ and used both a pre-experience and a post-experience questionnaire. We found that heterogeneity was present among both the participants and the procedures analyzed. All of these studies were RCTs in which, differently from the intervention group, the control group did not have access to an AR device; while the remaining instructions and other materials (books and didactic lessons) were equal for the two groups.

All of these four studies reported that changes in perspective or judgment occurred in the groups of participants, concerning teaching and learning (Kirkpatrick evidence level 2B).

Regarding procedures, two studies analyzed central venous catheter placement,^14,18^ one study evaluated the lumbar puncture procedure^15^ and one investigated injection into the interfacetal joint.^17^ Although a diversity of issues were analyzed among these trials, the performance achieved through the technique was the main outcome in all of them. Regarding the populations investigated, the participants comprised respiratory therapists, sleep technicians, pre-medical and medical undergraduate students, emergency medicine and surgery residents and anesthesiologists. In three of the studies analyzed,^14,15,17^ it was concluded that AR could increase students’ skills in interventional radiology.

AR is used in a variety of areas of medicine and no systematic review or clinical trial has been carried out using a homogenous population. Because of the heterogeneous nature of the populations studied, different AR devices analyzed and different medical procedures used in these four RCTs, we did not perform any meta-analysis. Table 3 shows the quality assessment and risk of bias analysis conducted using the Cochrane Collaboration tool.

**Table 3:** Quality assessment/risk of bias analysis using the Cochrane Collaboration tool

	Underlying bias	Resource bias	Setting bias	Educational bias	Content bias
Huang et al.^14^	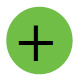	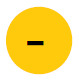	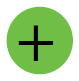	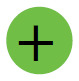	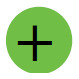
Keri et al.^15^	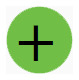	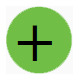	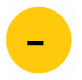	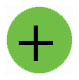	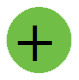
Moult et al.^17^	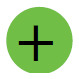	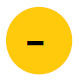	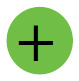	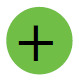	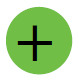
Wu et al.^18^	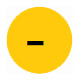	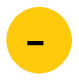	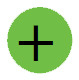	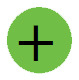	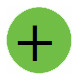

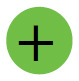
Low risk of bias.

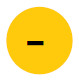
Unclear risk of bias.

Huang et al.^14^ enrolled 32 adult novice central line operators (physicians, respiratory therapists and sleep technicians) with no visual or auditory impairments. Comparisons were made between simulations using AR reality glasses and conventional instruction; the AR glasses used were Brother AiRScouter WD-200B AR glasses (Brother International Corp., Bridgewater, New Jersey, United States). The authors did not comment on the cost of the teaching techniques. The AR simulation group undertook a five to ten-minute hands-on instructional course on the AR device; the mean time taken for AR head placement was 71 seconds. No significant difference in the median time taken for internal jugular cannulation or in the median total duration of the procedure was found between the groups. Most participants (71%; n = 23) were successful in cannulating the internal jugular upon the first attempt (12 in the AR group versus 11 in the non-AR group). A significant difference in adherence level between the two groups (22.9 ± 4.1 in the AR group versus 18.1 ± 6.3 in the non-AR group; η^2^ = 0.90; P = 0.003) was detected. In the post-exercise questionnaire for the AR group, more than 80% of the participants stated that the instrument did not cause any fatigue and was not too heavy to be uncomfortable. Nonetheless, 30% admitted that the equipment affected their action skills and that it was not easy to regulate. On the other hand, 94% reported that the hand, head and foot interactions were undemanding and 80% stated that the information presented on-screen was suitable and reacted fast enough.

In the study by Wu et al.,^18^ 20 medical students and 20 emergency medicine residents were compared with regard to learning central venous catheter positioning. All the participants watched a video explaining how to use Google Glass and how to place an internal jugular central venous access catheter under ultrasound guidance in a simulation task trainer. The participants were randomized into two groups: with and without Google Glass. The ultrasound machine setup was the same between the groups; the intervention group participants were asked to execute the procedure by viewing ultrasound images displayed on their Google Glass screen, while the control group executed the procedure by viewing ultrasound images shown on the ultrasound screen. The Google Glass group took longer to perform the procedure, with longer times spent looking at the patient and monitor and greater numbers of needle redirections, at both training levels (medical students and emergency medicine residents). This may have been due to unfamiliarity with Google Glass, thus requiring more attention throughout the procedure. The responses to the post-exercise questionnaire showed that the majority of the participants were not previously familiar with AR or with wearable computing technology (75% and 60%, respectively); however, 73% reported having some degree of knowledge about Google Glass. Nonetheless, 87% of the participants randomized to Google Glass reported that the instrument was comfortable to use for the procedure.

Keri et al.^15^ evaluated the effectiveness of Perk Tutor (GPS extension, Ultrasonix, Canada) in relation to a phantom, as a teaching method among anesthesiology and surgery residents for lumbar puncture procedures. Perk Tutor is a training platform that was designed to display ultrasound images along with real-time three-dimensional images, using wearable technology. The authors did not comment on the participants’ experience levels regarding AR. There were 24 participants, who were divided into two groups (ten anesthesiologists and two surgeons): Perk Tutor with phantom and phantom alone. All the participants received a short presentation on spinal anatomy, ultrasound basics and how to use the device. They were also trained to perform ultrasound-guided procedures on three different lumbar spine models. The participants were then tested using conventional ultrasound guidance on an abnormal spinal model that they had not previously seen, for ten minutes at most or until positive fluid backflow ws observed at the needle hub. The potential tissue damage, needle path in tissue, total duration of the procedure and time taken to insert the needle were measured. Eleven participants in the phantom group and all participants in the Perk Tutor with phantom group performed the task successfully. The potential for tissue lesion was significantly lower in the Perk Tutor with phantom group (39.7 [range 21.3-42.7] square centimeters (cm^2^) versus 128.3 [50.3-208.2] cm^2^). Moreover, the needle tissue path was shorter (426.0 [range 164.9-571.6] millimeters (mm) versus 629.7 [306.4-2,879.1] mm), as also was the time taken to insert the needle (30.3 [14.0-51.0] seconds (sec) versus 59.1 [26.0-136.2] sec). The total duration of the procedure was similar (203.8 [range 135.1-274.9] sec versus 266.9 [221.6-416.2] sec).

Moult et al.^17^ compared the performances of 26 pre-medical undergraduate students (with no prior needle insertion experience) in a task of injection into the interfacetal joint. Participants were divided equally into two groups: Perk Tutor with phantom and phantom only. The authors did not comment on the cost of the teaching techniques. Both groups received a ten-minute introductory class on anatomy, procedure, ultrasound image interpretation and needle handling techniques. Afterwards, both groups had a ten-minute practice session on ultrasound-guided facet joint injections on the phantom; the Perk Tutor group had access to ultrasound and Perk Tutor, while the phantom group only had access to the ultrasound machine. The Perk Tutor and phantom group had a mean success rate of 61.5%, while this rate was 38.5% in the phantom only group; the total duration of the procedure was longer in the phantom only group (73 ± 8 versus 66 ± 6 seconds). The total needle distance travel (inside and outside of the phantom body) was greater in the phantom only group (1803 ± 290 versus 1366 ± 185 mm), but the inside distance traveled was shorter (25 ± 3 versus 42 ± 16 mm) in this group. Moreover, within the phantom body, the needle tip time was greater in the Perk Tutor and phantom group (296 ± 45 seconds versus 243 ± 28 seconds).

All of these results are summarized in Table 4.^14,15,17,18^

**Table 4:** Summary of studies’ findings.

Study	Country	Design	Participants	Procedure	Intervention	Comparison	Results	Kirkpatrick
Huang et al.^14^	United States	Randomized clinical trial with pre and post-experience questionnaire	32 novice central line operators including physicians, respiratory therapists and sleep technicians	Positioning of the central venous catheter	Augmented reality glasses Brother AiRScouter WD-200B AR glasses 17 participants	No augmented reality glasses 15 participants	Participants who trained in AR needed fewer attempts to perform the procedure.	2B
Keri et al.^15^	Canada	Randomized clinical trial with pre-experience questionnaire	24 anesthesiology and surgery residents	Lumbar puncture	Perk Tutor + phantom 12 participants	Phantom only 12 participants	Participants who trained in AR injured less tissue and were quicker to insert the needle	2B
Moult et al.^17^	Canada	Randomized clinical trial with pre-experience questionnaire	26 pre-medical undergraduate students	Injection into interfacetal joint	Perk Tutor + phantom 13 participants	Phantom only 13 participants	Participants who trained in AR obtained much better results, especially regarding the total duration of the procedure.	2B
Wu et al.^18^	United States	Randomized clinical trial with pre and post-experience questionnaire	10 1^st^ and 10 4^th^ year medical students; 10 1^st^ year emergency residents and 10 3^rd^ year residents	Positioning of a central venous catheter	Google Glass 5 participants	Without Google Glass 5 participants	Glass users took longer to complete the procedure.	2B

## DISCUSSION

The objective of our study was to examine the current evidence on training using AR in interventional radiology and its performance, along with the impact of AR on educational outcomes and skills, and its main advantages, disadvantages and challenges during the teaching-learning process.

New teaching techniques such as virtual reality (VR), AR or mixed reality (MR) are being introduced in medical education.^5,32^ AR combines virtual and real-world through use of wearable technology that provides a live feed from computer workstations (i.e. from an ultrasound device).^18^ Images and information are shown in the user’s line of sight through the device.^18^

Everyday use of mobile devices facilitates implementation of this instructional tool in teaching processes, which permits access to learning at any moment.^33,34^ However there is still a lack of research regarding the competence of this technology.^33^

AR methods have stood out in the surgical environment over recent years, through providing educational simulation practice free from potential ethical/hygiene concerns.^35^ Furthermore, the pressure imposed on healthcare systems during the COVID-19 pandemic has hastened implementation of new technologies, thereby accelerating the learning of healthcare professionals.^36^

Students are now used to dealing with technologies such as the internet, 3D video games, cell phones and others.^19,20,37–40^ Teachers can avail themselves of this familiarity to upgrade teaching methods and aids, so as to encourage students.^19,37,41^ Kotcherlakota et al. evaluated the utility of clinical simulation through applying AR technology to education outcomes for nurse practitioners in pediatric asthma management.^42^ The students showed high motivation, satisfaction and confidence scores.^42^ A systematic review by Barteit et al., on AR, VR and MR in several medical specialties, showed similar outcomes that revealed that these techniques were at least not inferior to traditional teaching methods.^43^ Moreover, these technologies offer opportunities for scalability and repetition without risk to patients.^43^

A systematic review by Rad et al. demonstrated that, in thoracic surgery, AR-enhanced intraoperative knowledge of anatomy decreased preoperative preparation time and workload.^35^ However, with regard to anatomy education, Bölek et al. concluded from a meta-analysis on five studies with a total of 508 participants that AR did not have any meaningful advantages or disadvantages for students’ education, compared with several traditional educational tools.^44^

AR could form a viable tool within traditional anatomy teaching in more technological environments.^44^ Küçük et al. found that neuroanatomy learning using AR with a smartphone provided support for students, through reducing cognitive effort and increasing educational pleasure.^19^ According to our systematic review, the results regarding AR are similar in several medical specialties.

The main purpose of AR involves the concept of “practice makes perfection”, given that efficient performance in procedures requires experience.^45^ AR simulation provides the possibility of repetition to boost self-confidence, within a safe method.^46^

Over five million central venous catheters are fitted each year in the United States. The complication rates are 5%-8% higher per procedure when these are handled by novice professionals.^14^ Teaching with AR aids could result in lower morbidity, hospitalization time and costs.

Comparison of learning between novice physicians and experienced interventional radiologists could enable evaluation of whether AR has the capacity to accelerate learning. Studies comparing different kinds of AR in one specified procedure need to be performed in order to determine which technology is better for that particular procedure. From the current information available, AR is a useful additional tool for teaching interventional radiology, but not a substitute for the traditional methodology.

From the students’ perspective, AR can contribute to mastery and confidence in a new procedure, through enabling students to memorize details, thus decreasing the tension in real-life situations. Regarding classroom ambience, AR may enable a shift from the monotonous routine of expository classes, thus providing evolution of the learning experience. Assembling education with technology would engage young people, thereby transforming learning into a pleasant experience and improving learning, as well as clinical practice.

One limitation of this systematic review was that only two studies analyzed the same procedure.^14,18^ Numerous procedures are involved in interventional radiology, but in the four studies evaluated, only three different procedures were investigated: central venous catheter placement, lumbar punctures and interfacetal joint injection. Two different types of AR devices were tested: Perk Tutor and AR glasses. Different AR devices could be compared in the future. Moreover, the small samples used in the studies represented another limitation, thus hampering generalization.

Another limitation was the lack of evaluation among experienced professionals. The participants included in these studies were novice physicians or non-physicians; none of these studies investigated radiology residents.

The level of evidence of the studies was also a limitation: all of them were classified as 2B in the Kirkpatrick model.^29^ Our searches did not retrieve any studies with educational evidence at level 3 (behavioral change), 4A (change in the organizational system/practice) or 4B (change among participants, students, residents or colleagues). Despite the current interest^22^ in using simulators, it remains to be delineated which types of simulation and simulator should be used, and what population this teaching method will be applied to. Hence, a higher level of evidence is needed.

Hardware needs are also a concern, considering that running the application produced intense energy usage as well as device heating.^34^ These technical difficulties could be resolved by the smartphone industry. Use of faster networks enables a shared environment through cloud services and shared real-time information. Introduction of artificial intelligence to AR-based learning programs can also provide more positive learning.

The costs of AR devices are expected to decrease along with the evolution of production and increased market competition, thus bringing these technologies to low-income countries. Moreover, AR-based medical training could facilitate teaching for people with reading limitations and could also facilitate remote teaching.

## CONCLUSION

It was demonstrated through this study that AR, as a complementary tool, can add skills to learners and thus can improve the teaching-learning process. It needs to be noted that only level 2B studies were found in this systematic review and, thus, that a higher level of evidence is required. Moreover, comparison of beginner physicians and expert interventional radiologists could enable appraisal of the hastening of the learning curve through AR, as well as investigation of which set of AR tools is most adequate for each procedure.

## Appendix

**Annex 1:** The 11 questions in BEME GUIDE no. 11 are as follows:

1. Are the research question(s) or hypothesis clearly defined?
2. Is the group of participants appropriate for the study being carried out (number, characteristics, selection and homogeneity)?
3. Are the methods used (qualitative or quantitative) reliable and valid for the research question and context?
4. Did the participants drop out? Is the dropout rate below 50%? For questionnaire-based studies, is the response rate acceptable (60% or more)?
5. Have several factors/variables been removed or accounted for whenever possible?
6. Are the statistical methods or other methods of analyzing the results used appropriately?
7. Is it clear that the data justify the conclusions drawn?
8. Could the study be repeated by other researchers?
9. Does the study look forward in time (prospective) and not backward (retrospective)?
10. Have all relevant ethical issues been addressed?
11. Were the results supported by data from more than one source?
